# Sustained Drug–Drug Interaction Between Cyclosporine and Apalutamide in a Patient With Metastatic Hormone‐Sensitive Prostate Cancer: A Case Report and Evaluation of CYP3A4 Induction via Pregnane X Receptor Activation by Apalutamide

**DOI:** 10.1155/crom/3539500

**Published:** 2026-02-24

**Authors:** Yoshihisa Mimura, Takaomi Sanda, Rei Unno, Yuki Furukawa, Ryota Shizu, Kotona Motokatsu, Yuusuke Tashiro, Yoshihiko Tasaki, Yosuke Sugiyama, Tomoya Yasujima, Taku Naiki, Yasuhiro Horita, Yuji Hotta, Hiroaki Yuasa, Kouichi Yoshinari, Takahiro Yasui, Shinsuke Iida, Yoko Furukawa-Hibi

**Affiliations:** ^1^ Department of Clinical Pharmaceutics, Nagoya City University Graduate School of Medical Sciences, Nagoya, Aichi, Japan, nagoya-cu.ac.jp; ^2^ Department of Hematology and Oncology, Nagoya City University Graduate School of Medical Sciences, Nagoya, Aichi, Japan, nagoya-cu.ac.jp; ^3^ Department of Nephro-urology, Nagoya City University Graduate School of Medical Sciences, Nagoya, Aichi, Japan, nagoya-cu.ac.jp; ^4^ Laboratory of Molecular Toxicology, School of Pharmaceutical Sciences, University of Shizuoka, Shizuoka, Shizuoka, Japan, u-shizuoka-ken.ac.jp; ^5^ Department of Biopharmaceutics, Nagoya City University Graduate School of Pharmaceutical Sciences, Nagoya, Aichi, Japan

**Keywords:** apalutamide, cyclosporine, cytochrome P450 3A4, drug–drug interactions, pregnane X receptor

## Abstract

**Background:**

Apalutamide (Apa) is a key therapeutic agent for prostate cancer. Despite its efficacy, Apa is known to induce several drug‐metabolizing enzymes including cytochrome P450 3A4 (CYP3A4), raising concerns about drug–drug interactions (DDIs). This study reports a rare case of a sustained DDI between Apa and cyclosporine (CsA)—a CYP3A4 substrate—in a patient with metastatic hormone‐sensitive prostate cancer (mHSPC) and primary pure red cell aplasia (PRCA). We further investigated whether Apa could activate pregnane X receptor (PXR), a key nuclear receptor that regulates CYP3A4 expression.

**Methods:**

With informed consent, residual blood samples were used to measure serum Apa concentration. To assess the potential of Apa in activating PXR, a reporter gene assay and a CYP3A4 mRNA induction test were performed.

**Case Presentation:**

A 75‐year‐old man with mHSPC was treated with Apa and leuprorelin. He developed PRCA and was administered CsA (5 mg/kg/day) on Day 1. Despite a target trough concentration (*C*_trough_) of 150–200 ng/mL, the *C*_trough_ of CsA remained subtherapeutic (34 ng/mL on Day 5), even after dose escalation to 10 mg/kg/day (*C*_trough_: 66 ng/mL on Day 7), suspecting a DDI with Apa. On Day 8, Apa was discontinued and the CsA dosage was reduced to 5 mg/kg/day. The Apa concentrations measured on Days 13, 26, and 34 were 1.1, 0.15, and 0.07 *μ*g/mL, respectively, and the *C*_trough_ of CsA increased to 45, 82, and 134 ng/mL, respectively. In vitro experiments demonstrated that Apa was a strong activator of PXR and capable of inducing *CYP3A4*.

**Conclusion:**

Apa induced CYP3A4 via the PXR pathway, leading to a sustained DDI with CsA. Careful monitoring is necessary when Apa is coadministered with CYP3A4 substrates.

## 1. Introduction

The incidence of prostate cancer (PCa), which is the most common cancer among men in Japan, is increasing globally [1, 2]. PCa generally has a better prognosis than that of other malignancies, even with distant metastasis; its 5‐year survival rate is approximately 50%, and long‐term survival can be achieved with drug therapy [1–4]. However, because the incidence of PCa increases with age, patients with PCa are frequently subjected to polypharmacy [5]. An observational study by Appukkuttan et al. reported a median of seven concomitant medications for 1515 patients with PCa who received drug therapy [5], thus highlighting the increased risk of drug–drug interactions (DDIs) and the need for careful monitoring.

Apalutamide (Apa) is a key therapeutic agent used to treat PCa. During the Phase III TITAN trial, Apa significantly improved the prognosis of patients with metastatic hormone‐sensitive prostate cancer (mHSPC) [3]. However, Apa can induce several drug‐metabolizing enzymes and transporters, including cytochrome P450 (CYP) 3A4, as demonstrated by a Phase I study [6]. DDIs may occur between Apa and drugs that are substrates of these enzymes and transporters. However, real‐world data regarding DDIs involving Apa are limited, and the molecular mechanisms underlying the induction of these enzymes and transporters are not yet fully understood.

We describe a clinically significant reduction in the blood concentration of cyclosporine (CsA), a substrate for CYP3A4 [7], caused by the concomitant use of Apa for a patient with mHSPC and primary pure red cell aplasia (PRCA). In this study, we measured the blood concentrations of both Apa and CsA and monitored CsA levels. Furthermore, we investigated the effect of Apa on the pregnane X receptor (PXR), which is a key regulator of CYP3A4 transcription [8], by performing a reporter assay and a CYP3A4 mRNA induction test.

## 2. Case Presentation

### 2.1. Case Presentation

A 75‐year‐old Japanese male patient (height, 1.63 m; weight, 74.8 kg) presented with a high prostate‐specific antigen (PSA) level of 564.104 ng/mL and hemoglobin (Hb) concentration of 4.7 g/dL indicating severe anemia. Computed tomography and magnetic resonance imaging revealed lymph node and bone metastases. A prostate biopsy confirmed adenocarcinoma with a Gleason score of 8 (4 + 4). Accordingly, mHSPC was diagnosed, and treatment with Apa (240 mg daily) and leuprorelin (22.5 mg every 12 weeks) was initiated. Fifty days after starting treatment for mHSPC, the PSA level significantly decreased to 0.852 ng/mL; however, anemia persisted (Hb level, 5.9 g/dL), and his condition became dependent on red blood cell (RBC) transfusions. An evaluation by hematologists revealed a diagnosis of PRCA.

**Figure 1:**
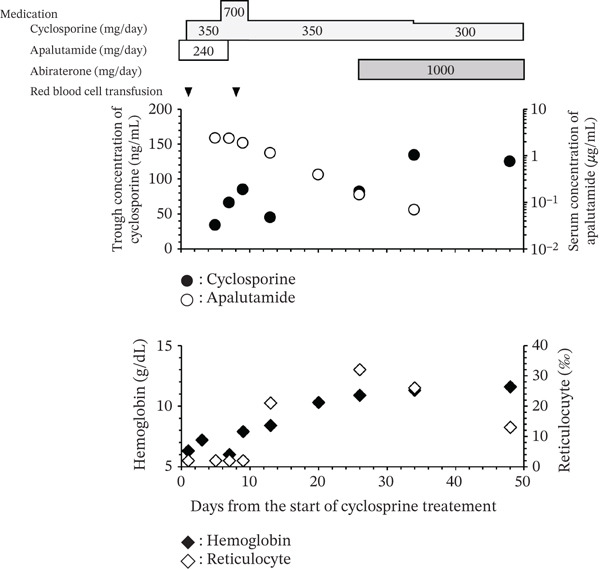
Schematic diagram of the treatment course. The *x*‐axis represents the number of days elapsed. Day 1 corresponds to the initiation of cyclosporine therapy. Black circles represent the trough concentration (*C*_trough_) of cyclosporine, which was measured on Days 5, 7, 9, 13, 26, 34, and 48. White circles represent the serum concentration of apalutamide measured on Days 5, 7, 9, 13, 26, and 34. Black triangles indicate red blood cell transfusions, which were performed on Days 1 and 8. Black squares represent the hemoglobin concentration measured on Days 1, 3, 7, 9, 13, 20, 26, 34, and 48. White squares represent the reticulocyte ratio assessed on Days 1, 5, 7, 9, 13, 20, 26, 34, and 48.

CsA, a key therapeutic agent for PRCA, was initiated on Day 1 at a dose of 350 mg (approximately 5 mg/kg/day), with a target trough concentration (*C*_trough_) of 150–250 ng/mL [9]. The blood test results on Day 1 indicated no objective organ disorders, including those of the hepatic or renal function (Supporting Information 1: Table S1). Figure 1 shows the timeline of this case, serum concentrations of Apa, and *C*_trough_ for CsA. The method used to measure the blood concentration of Apa is described in the Supporting Information section. On Day 5, the *C*_trough_ of CsA was 34 ng/mL; therefore, the CsA dose was increased to 700 mg/day (approximately 10 mg/kg/day). However, on Day 7, the *C*_trough_ of CsA increased to only 66 ng/mL. Hb and reticulocyte levels remained low and were accompanied by a low *C*_trough_ of CsA; therefore, RBC transfusions were required on Days 1 and 8, suggesting that CsA had insufficient efficacy. The patient had been administered magnesium oxide, sulfamethoxazole/trimethoprim, and olopatadine in addition to CsA and Apa. Among these treatments, only Apa reportedly induces CYP3A4 [6]. Therefore, we suspected that the low *C*_trough_ of CsA may have been caused by CYP3A4 induction by Apa. Because mHSPC was well controlled, as indicated by a PSA level of 0.852 ng/mL, and because further increases in the CsA dose were not feasible, Apa was discontinued on Day 8 and the CsA dose was reduced to 350 mg/day.

The Apa concentrations were 2.4 *μ*g/mL on Day 5 and 2.3 *μ*g/mL on Day 7, indicating that the serum concentration of Apa had reached a steady state. After its discontinuation, Apa levels gradually decreased to 1.8, 1.1, 0.39, 0.15, and 0.07 *μ*g/mL on Days 9, 13, 20, 26, and 34, respectively. Additionally, the *C*_trough_ of CsA increased substantially to 45, 82, and 134 ng/mL on Days 13, 28, and 34, respectively. Because the amylase level (138 U/L) was increased on Day 34, the CsA dose was reduced to 300 mg/day. This dose reduction resulted in a slightly decreased *C*_trough_ of 125 ng/mL on Day 48.

Reticulocytes, which are immature RBCs that indicate hematopoietic activity, began to increase from Day 13, and the Hb level was maintained without RBC transfusions. By Day 34, the *C*_trough_ of CsA reached the approximate target range, and the levels of reticulocytes and Hb increased to 26‰ and 11.3 g/dL, respectively. On Day 26, abiraterone acetate—which is an androgen biosynthesis inhibitor used as first‐line therapy for mHSPC [10] that reportedly does not induce CYP3A4 [11]—was initiated. Thereafter, both mHSPC and PRCA were well controlled, as indicated by a PSA level of < 0.003 ng/mL and an Hb level of approximately 13 g/dL.

### 2.2. Investigation of the Effect of Apa on PXR Activation and CYP3A4 Induction Using In Vitro Systems

PXR is a hepatic nuclear receptor that is activated by various chemicals and regulates the transcription of its target genes, including CYP3A and CYP2C subfamily genes [12]. Thus, PXR plays a central role in DDIs by inducing the expression of drug‐metabolizing enzymes. Several enzyme‐inducing drugs such as rifampicin and clotrimazole are known activators of PXR [13].

To evaluate the PXR‐activating potential of Apa, an in vitro reporter assay was performed using a human PXR expression plasmid and a reporter construct containing the PXR‐responsive element (dNR1). Apa treatment as well as rifampicin treatment increased reporter activity in a dose‐dependent manner, and their EC_50_ values were comparable. The EC_50_ values for Apa and rifampicin were 9.6 and 3.1 *μ*M, respectively, suggesting that Apa acts as a strong human PXR activator (Figure 2a).

Figure 2Effects of apalutamide (Apa) on pregnane X receptor (PXR) activation and *cytochrome P450 3A4 (CYP3A4)* induction in in vitro systems. (a) COS‐1 cells were transfected with expression plasmids for human PXR, a firefly luciferase reporter plasmid containing the PXR‐responsive element dNR1 under the control of the thymidine kinase promoter, and a Renilla luciferase control plasmid. After transfection, the cells were treated with vehicle (Veh; 0.1% dimethyl sulfoxide), Apa, or rifampicin at the indicated concentrations for 24 h. Firefly luciferase activity was measured and normalized to Renilla luciferase activity. The results are shown as relative reporter activity, with the Veh group set as 1. (b) HepaRG cells were treated with Veh (0.1% dimethyl sulfoxide), Apa, or rifampicin at the indicated concentrations for 24 h. *CYP3A4* mRNA levels were determined by a quantitative reverse transcription polymerase chain reaction. *CYP3A4* mRNA levels were normalized to those of *ACTB* mRNA to calculate relative mRNA levels. The results are shown as relative *CYP3A4* mRNA levels, with the Veh group designated as 1.

**Figure:**
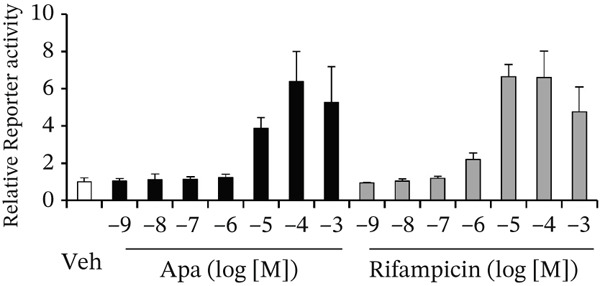
(a)

**Figure:**
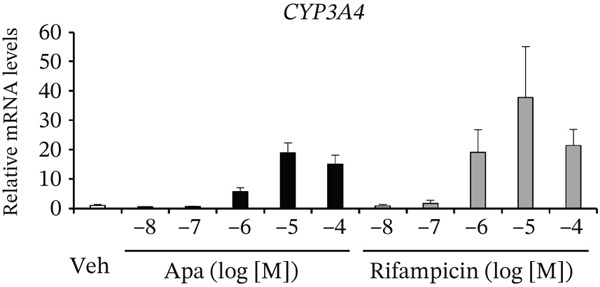
(b)

We also assessed the ability of Apa to induce CYP3A4 expression in human hepatocyte‐like HepaRG cells. Cells were treated with Apa or rifampicin, and CYP3A4 mRNA levels were determined using a quantitative reverse transcription PCR. The results revealed that both compounds, at concentrations as low as 1 *μ*M, increased CYP3A4 mRNA levels (Figure 2b). Together with the results of the reporter assays, these findings strongly suggest that, similar to rifampicin, Apa can induce CYP3A4 expression by activating PXR. Details of the reporter assay and the experiments using HepaRG cells are provided in the Supporting Information section.

## 3. Discussion

To the best of our knowledge, this is the first study to evaluate the relationship between the blood concentration of Apa and its potential for DDIs and investigate the mechanism underlying CYP3A4 induction by Apa using an in vitro model.

According to Phase I trials, Apa can induce various drug‐metabolizing enzymes and transporters, including CYP3A4, CYP2C9, CYP2C19, P‐glycoprotein, and breast cancer resistance protein, thus raising concerns regarding DDIs [6]. In particular, CYP3A4 induction is potent; for example, coadministration of midazolam, a typical substrate of CYP3A4, with Apa resulted in a 92% reduction in the area under the concentration–time curve and a 77% reduction in the maximum concentration. In our case, the *C*_trough_ of CsA, a substrate for CYP3A4 that is extensively metabolized in enterocytes and hepatocytes by CYP3A4 [7], decreased persistently, and the therapeutic efficacy was diminished. Furthermore, the findings of our case were consistent with those reported previously [14, 15] and support the notion that Apa may cause clinically relevant DDIs in real‐world settings.

A sustained reduction in CsA levels was observed even after Apa treatment was stopped. Apa has a long half‐life of approximately 4 days [6, 16]. In our case, Apa was discontinued on Day 7, and its serum concentration declined slowly, with a calculated half‐life of 122 h (approximately 5 days), consistent with the findings of previous studies [6, 16]. As the concentration of Apa decreased, the *C*_trough_ of CsA increased, suggesting that CYP3A4 induction by Apa was concentration‐dependent and long‐lasting, thus reflecting its long half‐life.

PXR is a nuclear receptor that regulates CYP3A4 expression [8]. Upon ligand binding, PXR translocates to the nucleus and regulates gene transcription. Our in vitro studies revealed that Apa was a strong human PXR activator capable of inducing *CYP3A4* expression. The EC_50_ value of Apa for PXR activation was calculated as 9.6 *μ*M, which is similar to that of rifampicin, a well‐known and strong human PXR activator, thus underscoring the strong potential of CYP3A4 to induce Apa. The EC_50_ of Apa calculated using an in vitro system (9.6 *μ*M is equivalent to approximately 4.6 *μ*g/mL) was comparable to its serum concentration of approximately 2.4 *μ*g/mL, which was the highest observed value. Interestingly, Apa exists in a highly protein‐bound state (approximately 90%) [17], and serum concentrations of the unbound form may be estimated to be an order of magnitude lower than the EC_50_ for PXR activation. Nevertheless, in the present study, the observed time‐dependent increase in the *C*_trough_ of CsA following Apa discontinuation suggests that CYP3A4 induction occurred even at relatively low serum concentrations of Apa. Further studies are warranted to elucidate this mechanism and improve the management of DDIs associated with Apa.

PRCA occurs at an incidence rate of 1.06 per million [18], and this particular case, associated with PCa, is very rare. However, the concomitant use of Apa with CYP3A4 substrates is generally more common. Patients with PCa tend to be older and have a high polypharmacy burden [5], and CYP3A4 is involved in the metabolism of more than 50% of clinically used drugs [19]. Many of these drugs do not have readily available therapeutic drug monitoring assays, and even CsA therapeutic drug monitoring is limited to specialized centers. Consequently, potential DDIs—such as that in the present case—may remain undetected. Therefore, healthcare professionals, including physicians and pharmacists, should remain vigilant of the strong and sustained CYP3A4‐inducing potential of Apa, as demonstrated in this study, and carefully monitor patients who are using Apa to identify DDIs. Particularly for CYP3A4 substrates with a narrow therapeutic index, such as certain immunosuppressants, anticoagulants, and antiarrhythmic agents, coadministration with Apa may result in subtherapeutic exposure and subsequent loss of disease control. Because these drugs are often managed in outpatient settings where therapeutic drug monitoring is not feasible, close clinical monitoring of the treatment response, disease activity, and potential signs of underexposure is warranted. Accordingly, when these drugs with a narrow therapeutic index must be combined with Apa, treatment modification—such as dose adjustment or switching either Apa or the concomitant medication—should be thoroughly discussed among the healthcare team.

In conclusion, we reported a rare case of a DDI between Apa and CsA. Our case and in vitro studies suggest that Apa can cause strong and sustained DDIs with CYP3A4 substrates, possibly through the activation of PXR. Therefore, healthcare providers should be aware of the potential of Apa to cause clinically significant DDIs. These findings highlight the importance of careful DDI management in patients with PCa, particularly when coadministering CYP3A4 substrates, and may contribute to safer and more effective treatment of PCa and its comorbidities.

## Funding

This work was the result of using research equipment shared in the MEXT Project for Promoting the Public Utilization of Advanced Research Infrastructure (Program for Supporting the Construction of Core Facilities) (grant number JPMXS0441500025.

## Ethics Statement

The authors have nothing to report.

## Consent

Informed consent was obtained from the patient for the publication of this case report.

## Conflicts of Interest

The authors declare no conflicts of interest.

## Supporting information


**Supporting Information** Additional supporting information can be found online in the Supporting Information section. Supporting materials and methods. Table S1: The result of the blood test at the start of cyclosporine therapy.

## Data Availability

The data that support the findings of this study are available on request from the corresponding author (Y.F‐H.). The data are not publicly available because they contain information that could compromise the privacy of the research participant.
