# Supplementary material for: Sustained Drug–Drug Interaction Between Cyclosporine and Apalutamide in a Patient With Metastatic Hormone‐Sensitive Prostate Cancer: A Case Report and Evaluation of CYP3A4 Induction via Pregnane X Receptor Activation by Apalutamide
Source: Case Rep Oncol Med. 2026 Feb 24;2026:3539500. doi: 10.1155/crom/3539500 (PMC12930205; doi:10.1155/crom/3539500)
Supplement: Supplementary file 1 — Supporting Information Additional supporting information can be found online in the Supporting Information section. Supporting materials and methods. Table S1: The result of the blood test at the start of cyclosporine therapy. [file CROM-2026-3539500-s001.docx]

**Supplemental Materials**

**1. Supplemental Materials and Methods**

***1-1. Informed consent***

Informed consent was obtained from the patient for the publication of this article and the accompanying blood test results.

***1-2. Measurement of cyclosporine and apalutamide concentrations***

Whole blood concentrations of CsA were measured as part of routine clinical practice (Abbott Japan, Chiba, Japan). Residual blood samples collected prior to each Apa administration were used to measure the serum Apa concentration. Measured serum Apa concentrations included both its protein-bound and unbound forms. The Apa concentration were analyzed using ultra-high-performance liquid chromatography tandem mass spectrometry (LCMS-8050; Shimadzu Corporation, Kyoto, Japan). After deproteinization using acetonitrile, the samples were processed using a Shim-pack Arata C18 column (Shimazu Corporation) in 0.1% formic acid in water and 0.1% formic acid in acetonitrile as a mobile phase with flow of 0.4 mL/min and subjected to liquid chromatography tandem mass spectrometry. Multiple reaction-monitoring transitions were recorded (Apa: m/z 478.1 > 161.95). Standard curves for Apa were constructed over the range from 0.01 µg/mL to 10 µg/mL. The correlation coefficient was > 0.999. Chromatographic data were processed using Lab Solution software (Shimadzu Corporation).

***1-3. Reporter assays***

COS-1 cells were seeded at a density of 1.0 × 10⁴ cells per well in 96-well plates using Dulbecco’s modified Eagle medium (Fujifilm Wako Pure Chemical, Osaka, Japan) supplemented with 10% fetal bovine serum (Cytiva, Tokyo, Japan), and cultured at 37°C in a 5% CO₂ atmosphere. Cells were transfected with the reporter plasmid dNR1-TK-pGL4.10, which was constructed by inserting four tandem repeats of the DR4 motif from the *CYP3A4* promoter (5’-GAATGAACTTGCTGACCCTCT-3’) into the Acc65I and BglII sites under the control of the thymidine kinase promoter (pGL4.10), human PXR expression plasmid (hPXR-pTarget), and *Renilla* luciferase-expressing plasmid (phRL-TK) using Lipofectamine 3000 (Thermo Fisher Scientific, Waltham, MA, USA) and treated with Apa or rifampicin, which is a representative human PXR agonist, in serum-free Dulbecco’s modified Eagle medium for 24 hours. Cells were lysed and reporter activity was measured using the dual-luciferase reporter assay system (Promega, Madison, WI, USA) according to the manufacturer’s instructions. Firefly luciferase luminescence was normalized to *Renilla* luciferase luminescence.

***1-4. Determination of CYP3A4 induction by apalutamide in HepaRG cells***

HepaRG cells (Biopredic, Paris, France) were seeded at a density of 3.6 × 10⁴ cells per well in collagen-coated half-area 96-well plates using Basal Hepatic Cell Medium supplemented with HepaRG^®^ Thaw, Seed, and General Purpose Supplement (Biopredic) and incubated at 37°C in a 5% CO₂ atmosphere. After 72 hours of incubation, cells were treated with Apa or rifampicin dissolved in Basal Hepatic Cell Medium supplemented with HepaRG^®^ serum-free induction supplement (Biopredic) for 24 hours. After treatment, the cells were washed with phosphate-buffered saline and lysed, and RNA was reverse-transcribed into cDNA using the SuperPrep II Cell Lysis and RT Kit for quantitative polymerase chain reactions (PCRs) (Toyobo, Osaka, Japan) according to the manufacturer’s instructions. Quantitative PCR was performed using the THUNDERBIRD^®^ Next Probe quantitative PCR Mix (Toyobo) with PrimeTime^®^ quantitative PCR assays (Integrated DNA Technologies, Coralville, IA, USA) for *CYP3A4* and *ACTB*. Reactions were performed using a StepOnePlus™ real-time PCR system (Applied Biosystems, Foster City, CA, USA).

***1-5. Calculation of half-maximal effective concentration value***

The half-maximal effective concentration (EC_50_) values were determined by performing a nonlinear regression analysis of concentration–response curves using iterative curve fitting (GraphPad Prism10; GraphPad Software, San Diego, CA, USA).

**2. Supplemental Table S1. The result of blood test at the start of cyclosporine therapy**

| Blood test | Result |
| --- | --- |
| WBC (×10^3^/µL) | 3.6 |
| Hb (g/dL) | 6.3 |
| PLT (×10^4^/µL) | 393 |
| Reticulocyte (‰) | 2 |
| Albumin (g/dL) | 3.2 |
| CRP (mg/dL) | 0.06 |
| AST (U/L) | 19 |
| ALT (U/L) | 17 |
| LDH (U/L) | 279 |
| ALP (U/L) | 108 |
| γ-GTP (U/L) | 29 |
| Creatinine (mg/dL) | 0.73 |
| BUN (mg/dL) | 15.4 |
| Total bilirubin (mg/dL) | 0.3 |

γ-GTP, gamma-glutamyl transpeptidase; ALP, alkaline phosphatase; ALT, alanine aminotransferase; AST, aspartate aminotransferase; BUN, blood urea nitrogen; CRP, C-reactive protein; Hb, hemoglobin; LDH, lactate dehydrogenase; PLT, platelet; WBC, white blood cell.
